# Urine metabolomic profiling of children with respiratory tract infections in the emergency department: a pilot study

**DOI:** 10.1186/s12879-016-1709-6

**Published:** 2016-08-22

**Authors:** Darryl J. Adamko, Erik Saude, Matthew Bear, Shana Regush, Joan L. Robinson

**Affiliations:** 1 The Department of Pediatrics, University of Alberta, T6G 1C9 Edmonton, Canada; 2 University of Saskatchewan, S7N 0W8 Saskatoon, Saskatchewan Canada; 3 Department of Emergency Medicine, University of Calgary, T2N 2T9 Calgary, Alberta Canada

**Keywords:** Metabolomics, Biomarkers, Bronchiolitis, Children, Respiratory syncytial virus, Bacteria

## Abstract

**Background:**

Clinicians lack objective tests to help determine the severity of bronchiolitis or to distinguish a viral from bacterial causes of respiratory distress. We hypothesized that children with respiratory syncytial virus (RSV) infection would have a different metabolomic profile compared to those with bacterial infection or healthy controls, and this might also vary with bronchiolitis severity.

**Methods:**

Clinical information and urine-based metabolomic data were collected from healthy age-matched children (*n* = 37) and those admitted to hospital with a proven infection (RSV *n* = 55; Non-RSV viral *n* = 16; bacterial *n* = 24). Nuclear magnetic resonance (NMR) measured 86 metabolites per urine sample. Partial least squares discriminant analysis (PLS-DA) was performed to create models of separation.

**Results:**

Using a combination of metabolites, a strong PLS-DA model (*R2* = 0.86, *Q2* = 0.76) was created differentiating healthy children from those with RSV infection. This model had over 90 % accuracy in classifying blinded infants with similar illness severity. Two other models differentiated length of hospitalization and viral versus bacterial infection.

**Conclusion:**

While the sample sizes remain small, this is the first report suggesting that metabolomic analysis of urine samples has the potential to become a diagnostic aid. Future studies with larger sample sizes are required to validate the utility of metabolomics in pediatric patients with respiratory distress.

## Background

In children, problems with breathing are the leading cause of hospitalization [1]. The most common cause of respiratory distress is airway obstruction secondary to viral bronchiolitis, most often due to respiratory syncytial virus (RSV) [1]. Each pulmonary disease entity requires different treatment, so correct diagnosis is important [2, 3]. Further, the severity of the disease determines the need for hospitalization. A test that could aid clinicians in determining the diagnosis and the severity of respiratory distress in young children in the emergency department would be of great benefit.

Metabolomics is the study of metabolic pathways and the unique biochemical molecules created in a living system [4, 5]. ^1^H–nuclear magnetic resonance (NMR) spectroscopy can be used to quantify specific chemical constituents within a body fluid [6]. NMR is an attractive technology because of its ability to non-invasively provide both qualitative and quantitative measurements, while simultaneously studying a number of compounds in the same biologic fluid. Urine is an excellent biological fluid for various medical studies owing to its ease of collection in patients of all ages, low cell and protein content, and rich chemical composition with over 1000 metabolites already identified by NMR [7].

Each airway disease has some differences in the type of airway cells involved. We hypothesized that children with RSV infection would have a different urine metabolomic profile compared to healthy controls, and that the severity of the respiratory disease would also cause changes in this metabolome. We also hypothesized that the urine metabolome of viral infection would differ from other causes of respiratory distress including bacterial infection.

In this report, we demonstrate for the first time that there is a specific metabolome associated with RSV-induced respiratory disease. We suggest that with further development, urine metabolomic data could become a useful non-invasive diagnostic aid for clinicians dealing with pediatric respiratory diseases.

## Methods

### Patient characteristics

Children of either gender and of any age admitted to the Stollery Children’s Hospital (Edmonton, Alberta) were approached for parental consent (as approved by the Health Research Ethics Board, University of Alberta) if they had any laboratory-proven viral or bacterial infection and the study nurse and parent were both available. Parents consented to the use of their child’s clinical data (e.g. age, sex, weight, vital signs, microbiology test results, and length of hospitalization) and collection of a urine sample. Some parents also agreed to follow-up urine sampling approximately two months later. Infections were considered to be proven if a virus was detected by antigen detection or culture from a nasopharyngeal sample or if bacteria were grown from blood or another sterile site. Children were excluded if co-infection was suspected or proven. A separate cohort of healthy control children was recruited through public health clinics while they attended their immunization visits (Table 1). A formal sample size calculation was not possible for this pilot study but a convenience sample of 100 children divided amongst the four groups (RSV, other viruses, bacterial infections, and controls) was thought to be sufficient to determine if further study of this potential diagnostic test is warranted.

**Table 1:** Patient Characteristics

	Age-matched (<2 years old)						Older children (>2 years)	
	Controls	Blinded Controls^a^	RSV	Blinded RSV^a^	Non-RSV Virus	Bacterial	Non-RSV Virus	Bacterial
	(*n* = 27)	(*n* = 10)	(*n* = 45)	(*n* = 10)	(*n* = 10)	(*n* = 14)	(*n* = 6)	(*n* = 10)
Median Age (months), Range	10.1 8.8–11.5	11.3 7.4–15.2	8.0 6.2–9.9	4.7 2.1–7.4	7.0 3.7–10.3	10.1 5.8–14.4	60 15.7–103.3	92.4 61.0–123.8
Sex (#Male/#Female)	28/12	22/11	25/20	5/5	5/5	5/9	6/0	8/2
Hospital Stay Mean # days (range)	N/A	N/A	5.2 (1–14)	7.2 (4–11)	7.8 (1–30)	19.6 (4–45)	8.6 (2–29)	18.3 (5–44)

^a^These subjects' samples were used for blinded analysis by the model to determine test accuracy

### Urine sample collection

It did not seem justifiable to obtain in and out catheterization samples. Therefore, urine bags were placed on the infants and removed as soon as possible after the child voided. In older children, midstream urines were collected. Samples were promptly placed in a freezer (−20 °C). Within 3 h of collection, the urine samples were stored in a −80 °C freezer. We have previously reported that such samples can be stable in the freezer for one year [8, 9]. We were aware of the variability in urine metabolomic data that can occur within each person related to diet and time of day [9, 10]; however our objective was to design a test that could be used in a typical clinical setting. Thus, we did not stipulate a specific time of day for each urine collection nor did we mandate dietary restrictions. We hypothesized that the metabolites of interest would be altered sufficiently between disease and non-disease groups that such intrapersonal variability would be superseded, as shown in our previous publications [11–13].

#### Statistical analysis of clinical data

Baseline characteristics were compared. As expected, the RSV group was younger than all other groups. To ensure that age was not a factor in the metabolomic models, older children (>2 years of age) were removed from the modeling and a Kruskal-Wallis with Dunn’s multiple comparisons test was used to confirm the age-matching of each group of children (GraphPad prism® (V.6)). Data are presented as mean with 95 % confidence intervals. A *p* value of <0.05 was considered significant. Length of hospitalization (1 to 3 days, 4 to 7 days, and > 7 days), was used as a surrogate for severity of RSVs, and metabolomics compared for those in the three groups and for controls to attempt to derive a severity-of-illness model.

### Sample preparation

Urine samples were thawed only once in a biosafety fume hood and a 630 μl aliquot was removed and placed in a 1.5 ml Eppendorff tube followed by the addition of 70 μl of a reference buffer solution ((4.9 mM DSS (disodium-2, 2-dimethyl 2-silapentane-5-sulphonate) and 100 mM imidazole in D_2_O) Chenomx, Edmonton, AB). The pH of each sample was adjusted to 6.8 +/− 0.1 using HCl and NaOH before transferring an aliquot of 600 μl to a standard 5 mm glass NMR tube (Wilmad, NJ, USA).

### NMR acquisition

All ^1^H-NMR spectra were acquired on a 600 MHz VNMRS spectrometer (Varian Inc, Palo Alto, Ca.) equipped with a 5 mm inverse-proton (HX) probe with Z-axis gradient coil. One-dimensional ^1^H-NMR spectra utilizing the first increment of a two dimensional-^1^H,^1^H-NOESY were collected at 25 °C with a spectrum width of 7200Hz, 4 steady state scans preceding acquisition, and 32 transients were acquired for each spectrum and apodized with an exponential decay corresponding to a line broadening of 0.5Hz prior to Fourier Transformation. Acquisition time per scan was 4 s, with a 990 ms saturation delay (power of 6) followed by two 90° pulses and a 100 ms mixing time and a final read 90° pulse. (detailed NMR acquisition parameters as previously described [12]).

### Spectral and statistical analysis

**Fig. 1:**
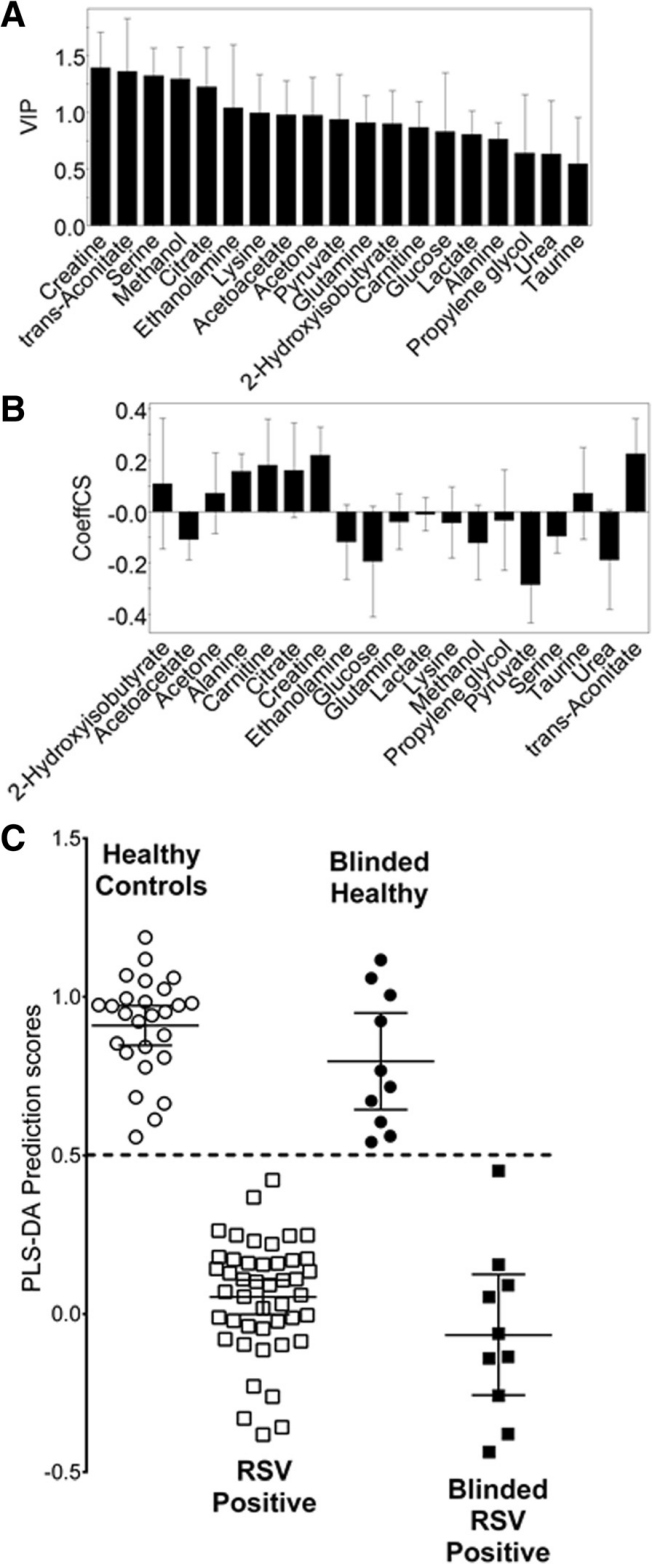
The metabolomic model of RSV bronchiolitis versus healthy control children. Urine metabolite levels were measured in age matched healthy children and compared to those with RSV bronchiolitis in the ED. Using a blind test set, PLS-DA analysis (SIMCA P-11) of these metabolites created a model of separation (*R2* = 0.86, *Q2* = 0.76). Illustrated are: **a** the Variables of Importance plot ranking the metabolites according to their significance in the model; **b** scaled and centered metabolite differences between groups shown as the Coefficients of Variation plot; **c** the PLS-DA prediction scores for each subject with error bars representing means and 95 % confidence intervals. The PLS-DA algorithm separates groups of data based on a score of 0–1; in this case a value above 0.5 indicates the infant is healthy while below 0.5 indicates RSV bronchiolitis

Spectral identification and quantification of 86 identifiable metabolites was performed using the Chenomx NMR Suite Professional software package Version 4.6 (Chenomx Inc., Edmonton, AB). The software contains a database of known metabolites with their referenced spectral resonant frequencies or signatures. These known resonant frequencies were matched to the observed resonant frequencies of the collected spectra, enabling the qualitative and quantitative analysis of metabolites in urine. To account for hydration status of the subjects, metabolites were referenced to creatinine and the values were log-scaled for normalization before Partial Least Squares Discriminant Analysis (PLS-DA) (SIMCA-P 11, Umetrics, USA). The SIMCA program performs seven-fold internal cross validation. This process identifies the metabolites whose concentrations differed significantly between groups of patients. As might be expected, most metabolites do not differ greatly between groups and including metabolites of low significance is detrimental to creating an accurate diagnostic model of separation. Metabolites with consistently greater difference in concentration between groups are displayed by the software as a Variables of Importance Plot (VIP) and a Co-efficient of Variation (COV) Plot (Fig. 1). To choose the most accurate list of metabolites, we removed metabolites listed as being lower in significance on the VIP until we were satisfied that the model could still correctly diagnose blinded samples not part of the model (10 healthy and 10 RSV positive infants). We set a false positive rate of 5–10 % as an acceptable limit. This resulted in the most sensitive models, both with respect to correlation coefficients (R2) and prediction properties (Q2). We previously reported using this technique in an animal model of asthma [11] and humans with asthma [12, 13]. The PLS-DA based model generates a prediction score (0–1) of unclassified, blinded data not part of the model (i.e., for data in Fig. 1 scores <0.5 are predicted to be RSV infected versus >0.5 are healthy controls).

## Results

### Baseline characteristics

Children enrolled included 55 with RSV, 16 with viruses other than RSV (primarily respiratory viruses), 24 with bacterial infections and 37 controls. The RSV group tended to be younger than the other groups. To ensure that age was not a factor in the metabolomic models, the comparison groups were age-matched as discussed in the Methods. As such, there was no significant difference in age for the models comparing RSV and non-RSV groups as shown in Table 1. Metabolomic data from the excluded older children (age >2 years) were still saved for comparison to the younger RSV-infected children and their data regarding age and sexes are shown in Table 1. More boys than girls were consented for study in the healthy control cohort. Duration of hospital stay for all groups is shown in Table 1. The etiologies of infection and number of children with each infection are outlined in Table 2.

**Table 2:** Non-RSV etiology of infection (# cases)

Age matched		Older children	
Parainfluenza virus	5	Parainfluenza virus	2
Influenza A	3	Influenza B	2
Influenza B	1	Influenza A	1
Adenovirus	1	Adenovirus	1
Streptococcus species	6	*Staphylococcus aureus*	5
*Staphylococcus aureus*	3	Streptococcus species	4
*Escherichia coli*	1	*Brucella melitensis*	1
*Enterococcus faecalis*	1		
*Klebsiella oxytoca*	1		
*Neisseria meningitis*	1		
*Stenotrophomonas*	1		

### Urine metabolomics can differentiate healthy controls from admitted patients with RSV

We compared the metabolites of age matched healthy controls (*n* = 27) to children with RSV infection (*n* = 45). As would be expected, most of the metabolites excreted in the urine did not differ greatly between groups, and adding metabolites of low importance rendered the PLS-DA based model less accurate. To remove irrelevant metabolites, we randomly withheld urine samples from 20 children (10 healthy and 10 RSV infected children) to be used as a test set. Using the test set, we removed metabolites that allowed the model to correctly classify these blinded patients. The final list of remaining metabolites used three components consisting of 19 metabolites in the VIP list giving an *R2* = 0.86, *Q2* = 0.76. The importance of the metabolites used for separation of these two groups is shown as a VIP Plot (Fig. 1a). The differences in concentration of these metabolites between groups are shown as the COV Plot (Fig. 1b). Graphic presentation of the quality of separation between groups in the model is shown by their respective PLS-DA scores (Fig. 1c). The final metabolites chosen and their concentrations are shown in Table 3.

**Table 3:** The concentration of each metabolite for each subject group is shown as the median and interquartile range (IQR) in mmol of metabolite/mmol creatinine

	Healthy Control Children			RSV			Other Respiratory Viruses			Other Respiratory Viruses			Bacterial Infection			Bacterial Infection		
							(Age-matched)			(Older children)			(Age-matched)			(Older children)		
	Median	IQR		Median	IQR		Median	IQR		Median	IQR		Median	IQR		Median	IQR	
2-Hydroxyisobuterate (α)^a^	0.014	0.012	0.017	0.008	0.004	0.013	0.006	0.004	0.011	0.006	0.003	0.011	0.007	0.002	0.013	0.011	0.005	0.020
3-Hydroxyisovalerate (β)^a^	0.019	0.010	0.028	0.014	0.010	0.020	0.014	0.004	0.021	0.023	0.011	0.036	0.012	0.004	0.021	0.015	0.009	0.022
3-Indoxylsulfate (β)^a^	0.018	0.006	0.052	0.022	0.006	0.057	0.026	0.006	0.061	0.031	0.009	0.097	0.006	0.006	0.015	0.012	0.006	0.028
Acetoacetate (α,β)^a^	0.004	0.003	0.010	0.012	0.006	0.020	0.016	0.008	0.038	0.023	0.008	0.063	0.010	0.005	0.029	0.017	0.005	0.034
Acetone (α)^a^	0.006	0.006	0.009	0.013	0.007	0.027	0.010	0.006	0.029	0.008	0.004	0.035	0.011	0.005	0.047	0.011	0.003	0.044
Alanine (α)^a^	0.131	0.104	0.164	0.156	0.090	0.230	0.140	0.057	0.239	0.080	0.023	0.155	0.113	0.076	0.193	0.044	0.014	0.091
Betaine (β)^a^	0.205	0.094	0.514	0.425	0.058	0.932	0.171	0.036	0.424	0.010	0.007	0.223	0.044	0.014	0.178	0.013	0.008	0.018
Blue 1.06 (β)	2.165	1.675	2.954	1.967	1.520	2.276	2.040	1.531	2.352	1.231	0.393	2.131	1.880	0.200	2.620	1.033	0.832	1.797
Carnitine (α)^a^	0.037	0.013	0.066	0.014	0.008	0.039	0.013	0.006	0.051	0.017	0.010	0.041	0.009	0.004	0.022	0.013	0.007	0.033
Citrate (α)^a^	0.690	0.485	1.089	0.334	0.182	0.602	0.153	0.093	0.540	0.251	0.053	0.615	0.197	0.041	0.402	0.183	0.086	0.399
Creatine (α)^a^	0.513	0.251	1.082	0.029	0.015	0.206	0.125	0.035	0.725	0.031	0.021	0.259	0.039	0.017	0.195	0.017	0.008	0.035
Ethanolamine (α,β)^a^	0.084	0.069	0.107	0.126	0.098	0.181	0.096	0.072	0.155	0.086	0.051	0.169	0.112	0.081	0.148	0.065	0.041	0.085
Fumarate (α,β)^a^	0.003	0.001	0.006	0.006	0.001	0.010	0.002	0.001	0.008	0.002	0.002	0.349	0.001	0.001	0.004	0.001	0.001	0.001
Glucose (α)^a^	0.096	0.067	0.136	0.170	0.103	0.258	0.189	0.145	0.217	0.066	0.031	0.191	0.293	0.115	0.907	0.062	0.034	0.156
Glutamate (β)^a^	0.010	0.010	0.010	0.037	0.010	0.076	0.010	0.010	0.066	0.010	0.010	0.087	0.010	0.010	0.044	0.013	0.010	0.064
Glutamine (α)^a^	0.139	0.112	0.153	0.215	0.140	0.337	0.187	0.134	0.295	0.218	0.076	0.529	0.188	0.077	0.378	0.052	0.022	0.191
Hippurate (β)^a^	0.160	0.083	0.292	0.104	0.038	0.219	0.137	0.038	0.290	0.236	0.111	0.464	0.538	0.103	0.992	0.173	0.070	0.306
Hypoxanthine (α)^a^	0.015	0.009	0.023	0.019	0.008	0.024	0.019	0.002	0.027	0.010	0.008	0.018	0.006	0.002	0.030	0.013	0.006	0.017
Lactate (α)^a^	0.032	0.026	0.047	0.052	0.037	0.074	0.057	0.043	0.084	0.030	0.017	0.049	0.051	0.040	0.075	0.012	0.011	0.038
Lysine (α)^a^	0.011	0.006	0.047	0.056	0.035	0.128	0.029	0.006	0.063	0.006	0.005	0.020	0.070	0.029	0.108	0.028	0.016	0.061
Methanol (α)^a^	0.001	0.001	0.008	0.014	0.008	0.018	0.012	0.008	0.020	0.002	0.001	0.008	0.012	0.009	0.036	0.006	0.003	0.011
N,N-Dimethylglycine (β)^a^	0.048	0.030	0.067	0.066	0.028	0.104	0.031	0.017	0.144	0.007	0.003	0.042	0.024	0.006	0.098	0.004	0.002	0.007
Pantothenate (β)	0.021	0.015	0.029	0.018	0.011	0.024	0.019	0.012	0.028	0.007	0.002	0.010	0.009	0.001	0.017	0.005	0.001	0.016
Propylene glycol (α)	0.018	0.002	0.089	0.053	0.019	0.136	0.073	0.017	0.172	0.017	0.008	0.045	0.068	0.018	0.336	0.024	0.007	0.084
Pyruvate (α)^a^	0.009	0.006	0.012	0.016	0.009	0.059	0.022	0.012	0.091	0.003	0.000	0.020	0.007	0.001	0.018	0.003	0.001	0.010
Serine (α,β)	0.075	0.020	0.123	0.197	0.146	0.282	0.179	0.020	0.281	0.020	0.014	0.148	0.264	0.102	0.302	0.035	0.020	0.208
Succinate (β)^a^	0.128	0.062	0.165	0.099	0.062	0.140	0.062	0.027	0.075	0.059	0.020	1.942	0.029	0.015	0.071	0.014	0.004	0.023
Tartrate (β)	0.008	0.001	0.016	0.018	0.005	0.030	0.012	0.001	0.026	0.003	0.001	0.051	0.004	0.001	0.030	0.001	0.001	0.002
Taurine (α)^a^	0.253	0.174	0.395	0.160	0.050	0.345	0.112	0.061	0.703	0.103	0.073	0.309	0.115	0.051	0.265	0.170	0.082	0.344
Threonine (β)	0.046	0.036	0.063	0.058	0.038	0.098	0.052	0.040	0.131	0.030	0.008	0.087	0.075	0.036	0.112	0.026	0.010	0.151
Uracil (β)^a^	0.009	0.001	0.016	0.013	0.001	0.018	0.011	0.001	0.024	0.007	0.004	16.34	0.001	0.001	0.026	0.001	0.001	0.011
Urea (α)^a^	33.60	23.80	49.10	33.20	22.90	50.67	50.83	23.36	73.76	27.94	12.87	40.72	47.22	24.11	83.14	39.75	28.99	47.18
trans-Aconitate (α)^a^	0.019	0.006	0.043	0.002	0.002	0.008	0.002	0.002	0.009	0.002	0.001	0.002	0.002	0.002	0.002	0.002	0.002	0.009

The metabolites used to discriminate the different groups of subjects are labeled as: (α) required for separation of Healthy Infants vs RSV infection; (β) required for separation of RSV vs. Bacteria. Metabolites labelled with^a^ are known to be endogenously produced within the human body

### Validation of the RSV versus control model

**Fig. 2:**
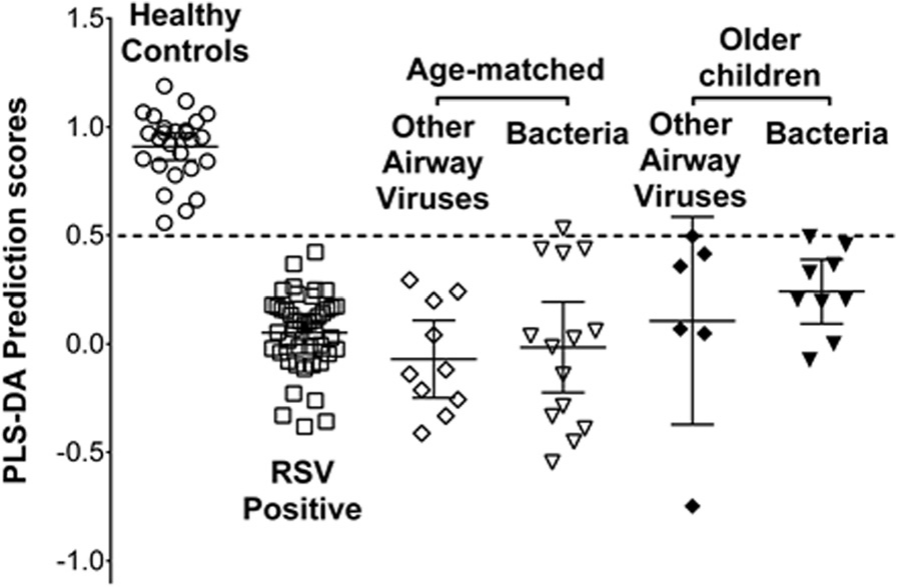
Validation of the model: prediction scores for children with other causes of respiratory distress. To determine some diagnostic accuracy, the metabolomic model of healthy children vs. RSV was presented blinded metabolomic data from children with respiratory distress from non-RSV infections. Illustrated are the PLS-DA prediction scores for each infant in the ED positive for either a non-RSV airway virus or bacterial infection. Error bars represent means and 95 % confidence intervals

Using this metabolomic model of RSV versus healthy control, we studied the other children (non-RSV respiratory virus, *n* = 16 and bacterial infection, *n* = 23). The values of the model’s specific metabolites were entered for these children and presented blindly to the PLS-DA based model to predict a diagnosis. We also stratified them based on being age matched (<2 years old) or being an older child (age > 2 years). The individual PLS-DA prediction scores are shown in Fig. 2 with error bars representing medians and interquartile ranges. The model perceived 97 % of these children (38/39) as having a metabolome more similar to the RSV group than to the controls. Age did not appear to be a significant factor in the data. The misclassified child had a *Staphylococcus aureus* infection post cardiac surgery.

**Fig. 3:**
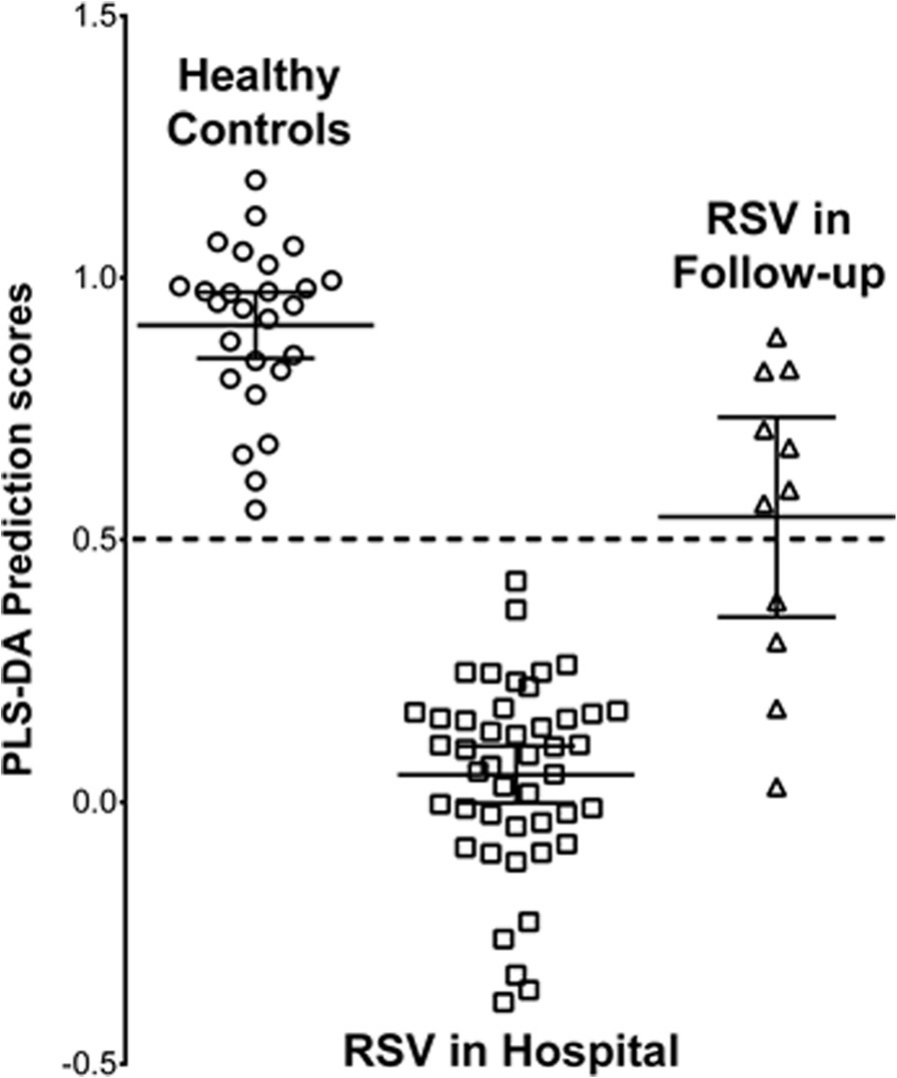
Prediction scores for children in follow-up post-RSV infection. To further determine some diagnostic accuracy, the metabolomic model of healthy children vs. RSV in the ED was presented blinded metabolomic data from RSV bronchiolitis children minimum 2 months post-infection. Illustrated are the PLS-DA prediction scores for each infant. Error bars represent means and 95 % confidence intervals

We also hypothesized that as children recovered their metabolome at the follow-up visit should also improve and become similar to healthy controls. Unfortunately, only 11 of the 46 RSV infected children had follow-up urines collected. The model perceived seven as healthy and four as having respiratory disease similar to RSV (Fig. 3). Unfortunately, we do not have any clinical data to determine whether these four had persistent symptoms of breathing difficulties in follow-up, which is a strong possibility.

### Urine metabolomic profile predicts RSV disease severity and length of hospitalization

**Fig. 4:**
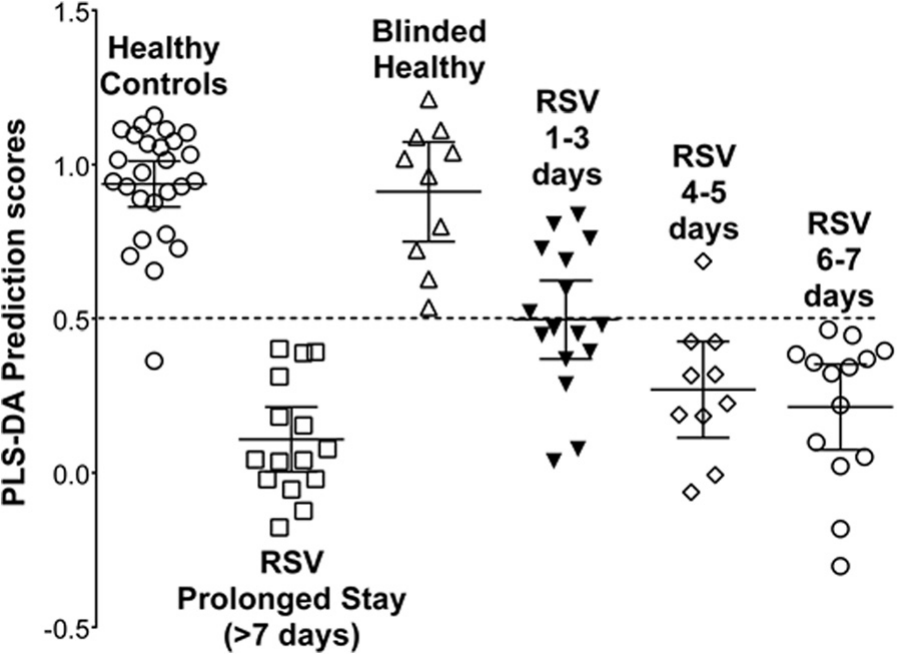
The metabolites used in the model of RSV bronchiolitis versus healthy control children also predict length of hospitalization. Using the same metabolites, we created a new PLS-DA-based model (*R2* = 0.83, *Q2* = 0.81) comparing the same healthy children in relation to the duration of hospitalization for RSV infection. Illustrated are the PLS-DA prediction scores for each infant stratified based on length of hospitalization. Error bars represent the means and 95 % confidence intervals

We stratified the children based on their length of hospital admission (long stay > 7 days; medium stay 4–7 days; short stay up to 3 days). We created a metabolomic model based on the ends of the spectrum, comparing healthy control children with those with RSV infection with long stays, using the same metabolites important for differentiating the healthy versus RSV infected children. We hypothesized that the value of these metabolites should reflect disease severity. Based on these metabolites, a model of separation was created (*R2* = 0.83 *Q2* = 0.81). Graphic presentation of the quality of separation between groups and the stratification by length of stay is shown by their respective PLS-DA scores (Fig. 4). There appears to be a step-wise change in the prediction scores based on duration of stay.

### Urine NMR metabolomic profiling to differentiate RSV infection from bacterial infection

We then determined if a metabolomic approach could discern differences between RSV infected children compared to those with non-RSV airway viral infection or compared to children with a bacterial infection. We first compared the metabolites of the RSV-infected children (*n* = 45) with those positive for a non-RSV respiratory virus (*n* = 16). There were no consistent differences in the 86 metabolites tested between RSV and non-RSV virus infection regardless of age, thus a significant PLS-DA model could not be generated.

**Fig. 5:**
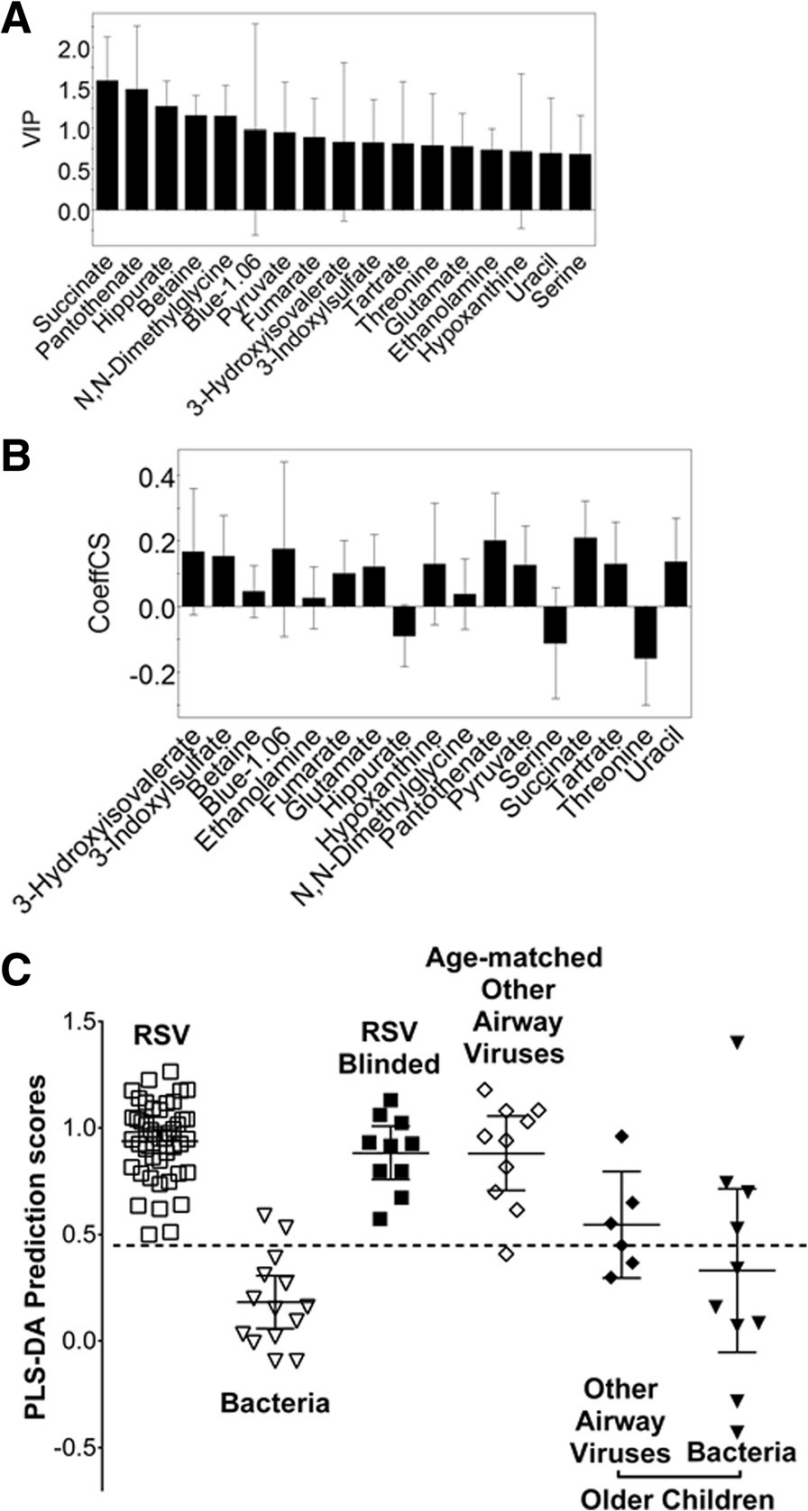
The metabolomic model of RSV infection versus age matched children with bacterial infection. Urine metabolite levels were measured in age matched children with bacterial infection and compared to those with RSV bronchiolitis. PLS-DA analysis of these metabolites created a model of separation (*R2* = 0.75, *Q2* = 0.61). Illustrated are: **a** the Variables of Importance plot ranking the metabolites according to their significance in the model; **b** scaled and centered metabolite differences between groups shown as the Coefficients of Variation plot; **c** the PLS-DA prediction scores for each subject with error bars representing means and 95 % confidence intervals. The PLS-DA algorithm separates groups of data based on a score of 0–1; in this case a value above 0.5 indicates the infant has RSV while below 0.5 indicates bacterial infection

In contrast, we compared the metabolites of RSV infected children (*n* = 45) with age-matched infants with a bacterial infection (*n* = 14). In this case, there were significant differences and a PLS-DA model could be generated. To remove irrelevant metabolites, we withheld 10 RSV-infected infants to be used as a test set. Unfortunately, we did not have enough age-matched bacterial infected infants to use as a test set. Thus, we removed metabolites that allowed the model to remain robust in terms of the R2Q2 value, while still correctly classifying the blinded RSV-infected children. The final list of remaining metabolites used two components consisting of 17 metabolites in the VIP list giving an *R2* = 0.75, *Q2* = 0.61. The importance of the metabolites used for separation of these two groups is shown as a VIP Plot (Fig. 5a). The differences in concentration of these metabolites between groups are shown as the COV Plot (Fig. 5b). Graphic presentation of the quality of separation between groups in the model is shown by their respective PLS-DA scores (Fig. 5c). The final metabolites chosen and their concentrations are shown in Table 3.

### Validation of the RSV versus bacteria model

Using this metabolomic model of RSV versus age-matched bacterial infants, we studied the other children (non-RSV respiratory virus, *n* = 16 and older children (>2 years of age) with bacterial infection, *n* = 10). We also stratified the non-RSV virus samples by age. The values of the model’s specific metabolites were entered for these children and presented blindly to the PLS-DA based model to predict a diagnosis. The individual PLS-DA prediction scores are shown in Fig. 5c. The model perceived 9 of the 10 age matched non-RSV samples correctly as a virus infected child (90 % correct). In contrast, samples from older children with non-RSV viral or bacterial infection were not perceived with high accuracy by this model. As such, age could be a significant factor in the data. Unfortunately, we did not have enough older children with RSV to create this model.

## Discussion

We have previously shown that metabolomic profiling of urine could differentiate children with asthma from healthy subjects and could differentiate stable from unstable asthma [12]. For the first time, we show that there are metabolomic differences in the urine of children with RSV infection that differ from healthy children and from children with bacterial infection, and also vary based on the severity of illness. At the time of manuscript submission, there were no articles found in a PubMed, Ovid and Embase literature search for urine based metabolomics studies of patients with respiratory viral infections. One article by Atzei [14] described metabolomics in neonates with RSV bronchiolitis, but they were still just in the early phase comparing urine collected from intubated and nonintubated infants.

While this was a pilot study, we have reviewed the metabolites in the context of their potential relation to known metabolomic pathways. Metabolites related to the citric acid cycle such as citrate, succinate and trans-aconitate appear to be important in subjects with viral respiratory tract infections. We previously documented similar citric acid cycle metabolites using an animal model of asthma [11]. Other researchers have hypothesized that citric acid cycle reflects stress and an increase in anaerobic glycolysis [15, 16].

Similarly, lactate, alanine, and acetoacetate are altered in a number of studies of inflammatory or metabolic perturbations. A metabolomics study of ventilator-induced lung injury in a rat model noted that in injured animals had higher concentrations of lung tissue as well as bronchoalveolar lavage lactate levels [17]. Increased lactate has been identified in metabolomics studies of pediatric asthma and have been suggested to reflect a stronger energy demand of allergic airway, abnormal lung respiration and altered energy metabolism, possibly under hypoxic or inflamed conditions [18]. Further, when lactate is produced, alanine levels are expected to change as well as part of gluconeogensesis [19]. Alanine levels were increased in the urine of piglet models of hypoxia Skappak [20], in the CSF of fetal hypoxic sheep [21] and cellular cultures undergoing apoptosis [22]. Perturbations in acetoacetate can be identified during periods of physical exertion and training in otherwise healthy individuals [23]. Animal studies of sepsis have noted that alterations in metabolites such as lactate, acetate and acetoacetate were identified in septic rats that did not survive [24, 25]. Increased serum levels of acetone have been related to increased myocardial energy expenditure in heart failure patients [26, 27]. Altered acetone levels were helpful in a model of exhaled breath condensate to differentiate healthy subjects from those with cystic fibrosis [28]. Many of these metabolites were similarly increased in infants with neonatal bacterial sepsis compared to healthy control infants [29]. The changes identified in our study surrounding acetoacetate, acetone and lactate may again point to altered energy metabolism in children with increased work of breathing secondary to respiratory viral infections.

Glutamine levels have been reported to be low in adults with chronic obstructive pulmonary disease (COPD) compared to healthy subjects [30, 31] and higher in asthma subjects compared to healthy controls [32]. Creatine has been reported in COPD patients in relation to work of breathing and muscle wasting [31]. Elevated levels of 3-hydroxyisobutyrate, acetone, alanine, and pyruvate have been correlated to early stages of cerebral ischemia and reperfusion injury models in rats [33]. Methanol was a component of the model to identify stable from unstable patients with cystic fibrosis [28]. A number of these same metabolites were found to be altered in our model and may point towards altered energy metabolism as well as ongoing injury to the lung with respiratory viral infections. None have been reported previously in children with RSV infection.

The majority of metabolomics research looking at ethanolamine has surrounded its involvement in the generation of cellular membranes and its utility in identifying apoptosis in cancer cells and possibly cellular respiration [34]. Ethanolamine has also been investigated as a source of carbon and nitrogen for bacterial during infectious pathogenesis and immune evasion [35]. Our analysis of urine from children with respiratory viral infections also detected perturbations in serine levels. Fluctuations in serine and ethanolamine and lipid layers have been noted in other pediatric respiratory diseases such as neonatal Respiratory Distress Syndrome. Further analysis is required to help elucidate the possible phospholipid changes that may be occurring in patients with viral respiratory tract infections.

We could find no publications specifically comparing differences in the metabolome of infants or children with viral versus bacterial infections. There was one study suggesting there are differences in adults with viral versus bacterial pneumonia, but the specific metabolites were not reported [36]. A group in Italy identified urinary metabolite perturbations in newborns with congenital cytomegalovirus infection. Our study of children with respiratory viral infections shared some similar metabolite changes; including taurine, betaine, and ethanolamine [37]. A metabolomic investigation of urine and plasma samples from children with severe pneumonia identified changes when compared with healthy controls [38]. Interestingly two of the metabolites identified in the Laiakis study [38], hypoxanthine and glutamate, were also found to be among the metabolites used to separate RSV infected children from those with bacterial infections in our current study. A mouse model of pneumonia due to either *Streptococcus pneumoniae* or *Staphylococcal aureus* noted changes in urine metabolites including ethanolamine, uracil, hippurate, betaine, succinate, fumarate, and 3-indoxysulfate [39], which were also important in our children with bacterial infection. Another metabolomics investigation of human urine sampled from patients infected with *Streptococcus pneumoniae* identified changes in fumarate, hypoxanthine, threonine, serine, and succinate [39]. Once again, these same metabolites were relevant in our model, distinguishing children with RSV versus bacterial infections. A unique feature of this present study is the ability to identify children with RSV confirmed respiratory infections as compared to those with a bacterial infection. Continued research in this area may advance the ability of metabolomics to impact the clinical identification and therefore care of these unique infectious etiologies.

There are limitations of the study. First, the sample size was small. We did not have enough age matched children for each category to fully validate the models. To still show some measure of accuracy, we used the infants (age matched) with non-RSV infection or bacterial infection. We also used this approach with the RSV versus bacterial differentiation. The data looked quite compelling when studying the blinded samples for potential accuracy. Second, bag urine samples could have been contaminated with bacteria. It did not seem justifiable to obtain catheterization samples for a pilot study. Urine bags were removed as soon as possible after the child voided and the urines frozen. In retrospect, it may have been useful to add an anti-bacterial to the urine bags to inhibit growth. Finally, the metabolite Blue 1.06 is an as yet unidentified metabolite. If this peak continues to show relevance in future work, we do plan to design methods using NMR and mass spectrometry to fully identify it.

## Conclusions

Overall, this was a pilot study intended to determine whether a metabolomic approach could be developed for use in the diagnosis and management of viral respiratory tract infections with an emphasis on RSV. In this report, we demonstrate for the first time that there is a specific metabolome associated with RSV-induced respiratory disease. This metabolome changes with the severity of illness and during recovery from illness. There also appears to be a metabolome differentiating respiratory distress from RSV compared to bacterial infection. Many of the metabolites identified were identified by previous work by our laboratory studying humans and animal with respiratory distress [11–13]. Additional model refinement and validation in on-going studies might help elucidate the principal metabolic pathways responsible for the unique pathophysiology observed in the pediatric lung and may better dictate management options for the bronchiolitic patient. The ultimate goal of this research is to develop a point-of-care test on urine that is both diagnostic and predictive of the severity of illness. This appears to be worth pursuing based on the results of the current study. As respiratory infections in young children lead to high hospitalization costs, improved diagnosis using a metabolomic test could lower health care costs by optimizing early management.

## Abbreviations

COV, coefficient of variation; ED, emergency department; NMR, nuclear magnetic resonance spectroscopy; PLS - DA, partial least squares discriminant analysis; RSV, respiratory syncytial virus; VIP, variables of importance plot
